# Comprehensive spatiotemporal evaluation of urban growth, surface urban heat island, and urban thermal conditions on Java island of Indonesia and implications for urban planning

**DOI:** 10.1016/j.heliyon.2024.e33708

**Published:** 2024-06-27

**Authors:** Faiz Rohman Fajary, Han Soo Lee, Tetsu Kubota, Vinayak Bhanage, Radyan Putra Pradana, Hideyo Nimiya, I Dewa Gede Arya Putra

**Affiliations:** a Transdisciplinary Science and Engineering Program, Graduate School of Advanced Science and Engineering, Hiroshima University, 1-5-1 Kagamiyama, Higashi-Hiroshima, 739-8529, Hiroshima, Japan; b Atmospheric Science Research Group, Faculty of Earth Science and Technology, Institut Teknologi Bandung, Bandung, 40132, West Java, Indonesia; c Center for Planetary Health and Innovation Science (PHIS), The IDEC Institute, Hiroshima University, 1-5-1 Kagamiyama, Higashi-Hiroshima, 739-8529, Hiroshima, Japan; d Graduate School of Science and Engineering, Kagoshima University, 1-21-40 Korimoto, 890-0065, Kagoshima, Japan; e Center for Research and Development, Indonesian Agency for Meteorology Climatology and Geophysics (BMKG), Jl. Angkasa 1 No. 2, Kec. Kemayoran, 10610, Jakarta, Indonesia

**Keywords:** Land use and land cover, Urban heat island, Land surface temperature, Urban thermal field variance index, Thermal discomfort, Remote sensing

## Abstract

Urban heat island (UHI) and thermal comfort conditions are among the impacts of urbanization, which have been extensively studied in most cities around the world. However, the comprehensive studies in Indonesia in the context of urbanization is still lacking. This study aimed to classify land use and land cover (LULC) and analyse urban growth and its effects on surface urban heat islands (SUHIs) and urban thermal conditions as well as contributing factors to SUHI intensity (SUHII) using remote sensing in the western part of Java Island and three focused urban areas: the Jakarta metropolitan area (JMA), the Bandung and Cimahi Municipalities (BC), and the Sukabumi Municipality (SKB). Landsat imagery from three years was used: 2000, 2009, and 2019. Three types of daytime SUHII were quantified, namely the SUHII of urban central area and two SUHIIs of urban sprawl area. In the last two decades, urban areas have grown by more than twice in JMA and SKB and nearly 1.5 times in BC. Along with the growth of the three cities, the SUHII in the urban central area has almost reached a magnitude of 6 °C in the last decade. Rates of land surface temperature change of the unchanged urban pixels have magnitudes of 0.25, 0.15, and 0.14 °C/year in JMA, SKB, and BC, respectively. The urban thermal field variance index (UTFVI) and discomfort index (DI) showed that the strongest SUHI effect was most prevalent in urban pixels and the regions were mostly in the *very hot* and *hot* categories. Anthropogenic heat flux and urban ratio have positive contributions to SUHII variation, while vegetation and water ratios are negative contributors to SUHII variation. For each city, the contributing factors have a unique magnitude that can be used to evaluate SUHII mitigation options.

## Highlights

• The SVM method provides more than 87 % of the overall accuracy.
• Since 2000, urban areas in the JMA and SKB have grown by over two times.
• In the last decades, SUHII UC almost reach 6 °C in the three cities.
• The rate of LST change of the unchanged urban pixels is quantified.
• The contributing factors have a unique magnitude for each city.

## Abbreviations

AH: Anthropogenic heat flux
B: Barren land
BC: Bandung and Cimahi cities/municiplaties
CCA: City clustering algorithm
DI: Discomfort index
DN: Digital number
DV: Dense vegetation/forest
ECMWF: The European Centre for Medium-Range Weather Forecasts
ENSO: El Niño-Southern Oscillation
ERA5: ECMWF Reanalysis v5
JMA: Jakarta metropolitan area
LST: Land surface temperature
LT: Local time
LULC: Land use and land cover
LV: Low vegetation
OLI/TIRS: Operational land imager/thermal infrared sensor
ONI: The Ocean Niño Index
PLS: Partial least square
sd-SUHII: Subdistrict level of SUHII
SI: Supplementary information
SKB: Sukabumi city
SUHI: Surface urban heat island
SUHII: Surface urban heat island intensity
SVM: Support vector machine
TM: Thematic mapper
TOA: Top of atmosphere
U: Urban
UC: Urban central
UHI: Urban heat island
UR: Urban ratio
US01: Urban sprawl 01
US02: Urban sprawl 02
UTFVI: Urban thermal field variance index
VR: Vegetation ratio
W: Water
WR: Water ratio

## 1 Introduction

Urban areas are currently home to more than half of the world's population and are expected to grow rapidly in the future [1,2]. Many people move to cities to improve their quality of life in terms of infrastructure, jobs, education, and other factors (economic growth and social development) [1,3,4]. Urbanization, however, can also generate many issues, and among those issues are environmental (ecological) aspects, such as air quality degradation (air pollution) and increases in thermal discomfort, energy use, and greenhouse gas emissions [[5], [6], [7]].

A well-known effect of urbanization is the appearance of the urban heat island (UHI) phenomenon, which is indicated by the elevated temperature of an urban area compared to its surroundings [8]. This phenomenon was first documented and published in 1818 by Luke Howard [9]. Many studies have been dedicated to analysing UHIs in various urban areas [[10], [11], [12], [13], [14], [15], [16], [17], [18]]. One type of UHI is surface UHI (SUHI), which characterizes the surface temperatures of the Earth, or objects built upon it. SUHI is mostly analysed using remote sensing to cover a wider area. The first SUHI study using remote sensing via satellite was reported by Rao [19] over the east coast of the U.S. Reviews of the use of thermal remote sensing in UHI studies are provided by many previous studies, such as Voogt and Oke [20]; Weng [21]; Mirzaei and Haghighat [22]; Chen et al. [23]; and Diem et al. [24], which include discussions of current issues and future research directions. Such issues include the difficulty in obtaining steady satellite images from urban surfaces, with one of the sources of this issue being cloud cover. Moreover, some portions of urban surfaces cannot be viewed due to the three-dimensional urban structure, preventing the vertical field of study.

Another effect of urbanization is related to thermal conditions, and two widely used indices to characterize them are the urban thermal field variance index (UTFVI) and the discomfort index (DI). The UTFVI is used to describe the UHI effect and quality of urban health and ecology due to its direct relation to land surface temperature (LST) [[25], [26], [27], [28], [29]]. The DI is used to examine human thermal comfort in several urban areas around the world [[30], [31], [32], [33], [34]].

As one of the developing and most densely populated countries, Indonesia has a high rate of urbanization in some areas, and Jakarta (the capital city of Indonesia) is classified as a megapolitan city (with a population of more than 10 million people) [35]. Some investigations have been conducted on land use and land cover (LULC) changes in Indonesia by using Landsat datasets in some cities, such as Jakarta [36,37] and the northern coastal region of West Java Province (Bekasi, Karawang, Subang, Indramayu, and Cirebon) [38]. Several studies have also investigated the impact of urbanization on SUHIs using satellite data, e.g., in Jakarta [39,40], West Java [41], and Semarang [42]. Compared to other regions, however, UHI studies in Indonesia are still limited as inferred from previous review papers [[43], [44], [45]], whereas Asian cities are the predominant target study areas (83.2 %) for studies on the relationship between SUHIs and LULC [45]. Moreover, most of UHI studies in Indonesia [[39], [40], [41], [42]] used a similar indicator (i.e., an area with LST higher than a specific threshold), and the analysis was carried out within city administrative boundaries or a single city. In fact, some cities have been developing beyond their administrative boundaries. By using that indicator, the area but not the intensity of the SUHI can be obtained. The intensity will be obtained by comparing LSTs between urban areas and reference areas, such as rural, agricultural, or water bodies [11,46,47]. Precisely estimating and analysing the intensity and amplitude of UHIs is necessary to develop a plan for better minimizing the effects of UHIs [48].

Furthermore, quantifying the factors that contribute to SUHI intensity (SUHII) is important due to the uniqueness of the causes of UHIs across different climates or city features [22]. Numerous studies have examined the various factors controlling the SUHII in different locations, such as in mid-latitude cities [49], major cities in China [50,51], and India [52]. Kurniati and Nitivattananon [53] revealed that greening, electricity consumption, and asphalt use are the significant factors in Surabaya, Indonesia. However, such studies in other Indonesian cities are still limited. By knowing recent urban growth and the SUHII and its contributing factors, mitigation measures can be effectively prioritized for sustainable urban planning, especially for developing countries, to achieve SDGs, in particular SDG11. Therefore, it is desirable to conduct a comprehensive study that involves the analysis of urbanization, its impact on variations in urban heat and thermal conditions, and factors that contribute to the SUHII variations. This study is the first comprehensive study on the characteristics of the thermal environment in the context of urbanization in Indonesia.

The purposes of this study are to classify LULC and analyse urban growth and its effects on UHIs and urban thermal conditions via remote sensing in the area with the highest population density in Indonesia, the western part of Java Island, where Jakarta is located. An additional goal is to quantify the factors contributing to the variation in the SUHII. This kind of comprehensive study is critical and will be useful to the government and stakeholders for sustainable urban development and for designing the new capital city with more sustainable schemes.

This article consists of five sections. Section 2 describes the study area, materials, and methods. Section 3 presents the results, highlighting two subsections: the first is LULC dynamics, urbanization, and LST; the second is the effects of urbanization on LST. Section 4 discusses the implications of this study for urban planning and the limitations of this study, as well as suggestions for future research. Finally, Section 5 summarizes the main findings.

## 2 Methodology

### 2.1 Study area

**Fig. 1:**
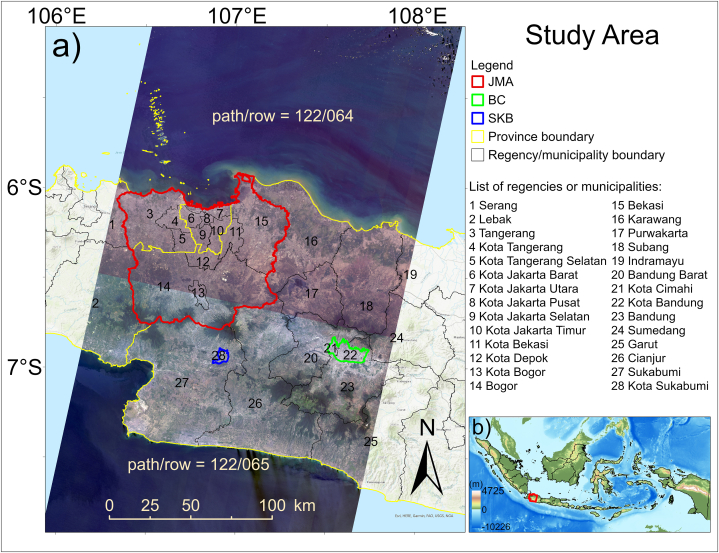
a) Landsat 8 OLI/TIRS collection 2 Level-2 images showing the study area (only over the mainland) in natural color (bands 4, 3, and 2 combination) with paths/rows 122/064 and 122/065 of Landsat scenes taken at instantaneous time on September 11, 2019. b) Location of the study area (red polygon) on the Indonesia map. The shading shows the topography.

The study area is covered by two paths/rows of Landsat scenes over the mainland, namely, 122/064 and 122/065 (Fig. 1). The geographical location of the study area is approximately 7°35′35″ S–5°53′58″ S in latitude and 106°7′54″ E−107°55′11″ E in longitude, and the elevation varies from 0 to approximately 2,957 m. According to the Köppen-Geiger classification, the climate of the area is mostly Af-type (tropical rainforest), with rainfall in the driest month more than or equal to 60 mm month^−1^. However, in the northern part, the climate is Am (tropical monsoon), while in the northern coastal area, the climate is Aw-type (tropical savannah). A characteristic of the tropical climate is that the air temperature in the coldest month is more than or equal to 18 °C. Moreover, in a limited area in the mountainous regions in the middle of the study area, the climate is Cfb-type (temperate, without a dry season) [54].

The study area includes three provinces, namely, Jakarta, some parts of West Java, and some parts of Banten (Fig. 1). These provinces are the top three most densely populated provinces in Indonesia, namely: 15,978 people/km^2^ for Jakarta; 1,379 people/km^2^ for West Java; and 1,248 people/km^2^ for Banten in 2021 [55]. Moreover, West Java Province is Indonesia's third largest rice producer [56]. There are a total of 15 regencies (Kabupaten in the Indonesian term) and 13 municipalities (Kota in the Indonesian term) in the study area. Regency and municipality in Indonesia have the same authority to control their territory, although a municipality is generally urbanized and serves as a center of economic activity [57].

The study area also covers the Jakarta metropolitan area (JMA) and the other remaining municipalities, namely, Bandung Municipality (the capital city of West Java Province), Cimahi Municipality, and Sukabumi Municipality. Jakarta, the capital and largest city of Indonesia, is a governmental and economic center and has experienced huge urbanization growth. Many people migrated to Jakarta, and the JMA was introduced administratively. The JMA consisted of Jakarta and its satellite cities, such as Bogor, Depok, Tangerang, and Bekasi [58]. Between Jakarta and Bandung, the region has been characterized by the formation of an urban belt of 200 km, which is growing to become a mega-urbanization region and playing an important role in the national economy [59]. In the west Java province, Bandung-Cimahi Cities (BC) is the region with the highest population density. Sukabumi City (SKB) is also a city with a high population density and growth rate in the province. This city is also surrounded by Sukabumi Regency, which has many conservation areas. Therefore, JMA, BC, and SKB were selected as the focus of the study for the three urban areas.

Although limited to three cities, our city samples encompass diverse characteristics. First, in terms of population, JMA, BC, and SKB, respectively, can be categorized into megapolitan (>10 million citizens), metropolitan (>1 million citizens), and medium cities (100,000–500,000 citizens) [35]. Secondly, in terms of area, JMA (6,783 km^2^), BC (209.31 km^2^), and SKB (48.33 km^2^) are categorized into large, medium, and small cities, respectively. Then, in terms of geographical background, JMA is a city located near the coast; BC and SKB are highland cities, with BC situated in a basin surrounded by mountains. Based on the urban growth patterns [60], JMA is suitably classified as a combination of "outlying" and "edge-expansion" growth because initially the city center and its satellite cities were developing and merging, forming a larger urban area. BC is more suitable in the "infilling" growth category as a basin-type city surrounded by mountains, so the horizontal outward expansion will be more limited, while SKB is more suitable in the "edge-expansion" category as it will experience extending outward.

Historically, Jakarta is an old city that was originally formed in the 15th century as a small port on the north coast of Java and is continuing to develop into one of the largest cities in the world today [61]. Bandung City was developed at the beginning of the 18th century after transportation hubs in the Java region were completed [62]. Meanwhile, Sukabumi City is a relatively new city that was formed in the early 20th century. Despite the limited sample size, the unique characteristics of these cities encompass a wide spectrum, offering insights into various cities across Indonesia and contributing to the originality of this study.

### 2.2 Materials

The main dataset used in this study is Landsat imagery (https://earthexplorer.usgs.gov/). The information about the satellite images used is shown in Table A.1. The data from three different years, namely, 2000, 2009, and 2019, were utilized. The images are from the Landsat 5 Thematic Mapper (TM) for 2000 and 2009 and the Landsat 8 Operational Land Imager/Thermal Infrared Sensor (OLI/TIRS) for 2019. Landsat 5 TM imagery consists of seven spectral bands with a spatial resolution of 30 m, except for thermal (band 6) collected at 120 m (resampled to 30 m). While Landsat 8 has eleven bands with spatial resolutions of 30 m (Bands 1–7 and 9), 15 m (Band 8), and 100 m (Band 10–11, resampled to 30 m) (https://www.usgs.gov/).

For LULC classification, Landsat Collection 2 Level-2 (C2 L2) was used, and for LST analysis, Landsat C2 L1 was used. Several images with different dates within the same season were utilized for LULC classification to complete the cloud removal. However, for LST retrieval and analysis, only one image for each path/row and each of the years was analysed, namely: September 6, 2000 (20000906), July 29, 2009 (20090729), and September 11, 2019 (20190911). These images have the least cloud cover, and there was no precipitation or atmospheric disturbances around the selected dates. The scene center time is only listed for the images with bold dates at approximately 3 UTC or 10 a.m. local time (LT).

The procedures for choosing the images were as follows. Several Landsat images with minimum cloud cover from June-July-August-September (dry season in the study area) from years *n* − 1, *n*, and *n* + 1 were collected as candidates (for example, for 2020, images for 2019, 2020, and 2021 were taken). In the end, the selected images were mostly from 2000, 2009, and 2019 (Table A.1).

In addition, the population density ($P_{den}$ in $\text{people}/km^{2}$) datasets for 2000, 2009, and 2019 were downloaded from WorldPop (www.worldpop.org) at a resolution of approximately 1 km at the equator and based on country totals adjusted to match the corresponding official United Nations population estimates [63]. Then, the following top-down approach [[64], [65], [66]] was used to calculate the anthropogenic heat flux (AH, $W/m^{2}$):

(1) $$AH\left(x,t\right)=P_{den}\left(x,t\right)\times E_{avg}\left(t\right)\text{.}$$

where the variables x and t denote space and time, respectively, and $E_{avg}\left(t\right)$ is the average energy consumption per person (W/person). In the case of Indonesia, $E_{avg}\left(2000\right)=731$, $E_{avg}\left(2009\right)=809$, and $E_{avg}\left(2019\right)=871\;W/\text{person}$, which are derived from the final energy consumption of multiple energy sources (such as electricity, biomass, coal, natural gas, oil fuel, briquette, LPG, etc.) with the dataset provided by the Ministry of Energy and Mineral Resources, Republic of Indonesia (the dataset is available in the SI).

Additional datasets, such as air temperature (t) and dew-point temperature ($t_{d}$), from the European Centre for Medium-Range Weather Forecasts (ECMWF) Reanalysis v5 (ERA5) are used. The data were downloaded from the Copernicus Climate Change Service [67]. Moreover, the topography and the administrative boundaries in shapefiles are provided by the Geospatial Information Agency of Indonesia and available on the web page (https://tanahair.indonesia.go.id/portal-web).

### 2.3 Methods

#### 2.3.1 Land use and land cover classification

For the LULC classification, multispectral bands were employed [68,69], six bands were used for Landsat 5 TM (bands 1–5 and 7), and seven bands were used for Landsat 8 OLI/TIRS (bands 1–7). Before performing the classification, the cloud covers of the best images (images with the minimum cloud cover and bold text dates in Table A.1) were removed, and then, they were filled by using another (the other) selected image(s) and simple interpolation. These images were projected by using the WGS84 datum with the geodetic system of the Universal Transverse Mercator (UTM) zone 48S.

Five class categories were used for classification, namely, urban (U), water (W), barren land (B), low vegetation (LV), and dense vegetation/forest (DV), as shown in Table 1. A supervised classification by a support vector machine (SVM) was used for the multispectral bands [[70], [71], [72], [73]]. A review paper by Li et al. [74] showed that SVM is one of the most extensively used methods for LULC classification. Before applying the method, the training samples for each LULC class were obtained by considering the higher resolution of the Google Earth pro images and the band combinations of the Landsat images (natural color, color infrared, false color, etc.).

**Table 1:** Classification categories of LULC and their descriptions, modified from previous studies [75,76]. Note that rice fields can be joined to either barren land or low vegetation depending on the plantation phase.

No	Classification categories	Description
1	Urban (U)	Building, residences, areas covered by asphalt and concrete, industrial, transportation, built-up areas
2	Water (W)	Ocean, coastal water, river, lakes, dams, ponds
3	Barren land (B)	Bare land surface, land with sparse vegetation, beaches, open land, rice field (after harvesting)
4	Low vegetation (LV)	Pastures, complex cultivation, cropland, green bush, rice field (during growing phase), tea farm
5	Dense vegetation/forest (DV)	Dense forest, mangrove forest, dense high trees including fruit tress (palm oil and coconut trees)

The classification accuracy was assessed by using a confusion or error matrix. It calculates the user's accuracy, producer's accuracy, overall accuracy, and Kappa coefficient [73,77,78]. Monserud and Leemans [79] characterized different ranges of the Kappa coefficient as follows. Values between 70 % and 85 % indicate very good agreement, values between 85 % and 99 % indicate excellent agreement, and values above 99 % indicate perfect agreement. Another study [80] reported very good agreement, with Kappa coefficient between 81 % and 100 %. The overall accuracy in identifying LULC categories from remote sensing data should be at least 85 % [81,82].

In this study, many random points (1,135 points) were generated, and the classification results and the reference dataset were subsequently compared via cross-tabulation. There was no overlap between the training samples and reference points. Consistent with prior research [69,[83], [84], [85]], in this study, reference data points were collected from geographic information systems (GIS) and on-site field surveys.

#### 2.3.2 Land surface temperature

Several calculation steps were performed to obtain the LST [86] as follows.

1) Top of atmosphere (TOA) spectral radiance

This equation is used to determine the spectral radiance for Landsat 5 using band 6 following Sultana and Satyanarayana [86] and other studies cited in them:

(2) $$L_{\lambda }=\frac{L_{\max}-L_{\min}}{{QCAL}_{\max}-{QCAL}_{\min}}\times \left(QCAL-{QCAL}_{\min}\right)+L_{\min}$$

where $L_{\lambda }$ is the TOA spectral radiance ($Wm^{-2}\text{sra}d^{-1}\mu m^{-1}$). QCAL (= DN or Digital Number) denotes quantized and calibrated standard product pixel values, with ${QCAL}_{\min}=1$ and ${QCAL}_{\max}=255$. $L_{\max}$ and $L_{\min}$ are the maximum and minimum spectral radiances, respectively, for band 6 at DNs 1 and 255, as provided in the metafile of the images.

The equation for Landsat 8 utilizing band 10, as in Avdan and Jovanovska [87], is:

(3) $$L_{\lambda }=M_{L}\times QCAL+A_{L}-O_{i}$$

where $M_{L}$ represents the band-specific multiplicative rescaling factor, QCAL is for the band 10 image, and $A_{L}$ is the band-specific additive rescaling factor. Both $M_{L}$ and $A_{L}$ are provided in the metadata file. $O_{i\;}$ is the correction value, with a magnitude of 0.29 ($Wm^{-2}\text{sra}d^{-1}\mu m^{-1}$) [88].

2) TOA brightness temperature

This variable is calculated using Eq. (4):

(4) $$T_{B}=\frac{K_{2}}{\ln\left(\frac{K_{1}}{L_{\lambda }}+1\right)}$$

where $T_{B\;}$ (K) represents the TOA brightness temperature. $K_{1}$ ($Wm^{-2}\text{sra}d^{-1}\mu m^{-1}$) and $K_{2}$ (K) are band-specific thermal conversion constants provided in the metafile.

3) LST estimation

The last step is LST calculation using Eq. (5):

(5) $$LST\left(K\right)=\frac{T_{B}}{1+w\frac{\;\sigma T_{B}}{hc\;}\ln\left(\epsilon \right)}$$

where w (μm) is the emitted radiance wavelength. For Landsat 5 and 8, w=11.5 [86] and w=10.895 [87,89], respectively. h is Plank's constant ($6.626\times 10^{-34}\;J\;s$), σ is the Boltzmann constant ($1.38\times 10^{-23\;}J\;K^{-1}$), and c is the speed of light ($2.998\times 10^{8}\;m\;s^{-1}$). $\epsilon =0.004\times P_{v}+0.986$ is the emissivity, and $P_{v}$ is defined as the proportion of vegetation according to the mathematical expression in Eq. (6):

(6) $$P_{v}={\left[\frac{NDVI-{NDVI}_{\min}}{{NDVI}_{\max}-{NDVI}_{\min}}\right]}^{2}$$

where NDVI=(Band4−Band3)/(Band4+Band3) for Landsat 5 and NDVI=(Band5−Band4)/(Band5+Band4) for Landsat 8. Finally, the LST is converted to °C by using Eq. (7):

(7) LST(°C)=LST(K)−273.15

#### 2.3.3 Surface urban heat island intensity

The SUHII is defined as the difference in LST between urban area and rural area. A city clustering algorithm (CCA) was adopted to delineate the urban area [[90], [91], [92]] instead of using the administrative city boundary. In CCA, a cluster parameter (*s*, number of cells) must be defined. The same urban cluster is assigned to any pair of urban cells separated by no more than the predefined cluster s. Previous studies have used different s, s=8 [90], s=2 [91], and s=1 [92] for different cities. In this study, s=2 was chosen from sensitivity tests because the clustered results approximate the actual spatial extent of the urban areas.

A rural area was defined as a buffer zone with a distance of 15–20 km [93] to the urban boundary of 2019 and excluding water and urban (impervious surface) pixels [[90], [91], [92]]. Any pixels with elevation beyond ±50 m of the urban mean elevation were also excluded [10,94] to suppress the interference of terrain in SUHII estimation. In this study, multiple static urban boundaries were also defined, namely urban central and two urban sprawl areas. Urban central (UC) is an area inside the urban boundary of 2000, while the first urban sprawl (US01) is a zone between the urban boundaries of 2009 and 2000, and the second urban sprawl (US02) is a zone between the urban boundaries of 2019 and 2009. The three types of SUHII in the three urban zones were also quantified: SUHII UC, SUHII US01, and SUHII US02.

#### 2.3.4 Land surface temperature of the unchanged urban pixels

Ideally, the impact of urbanization should be examined by comparing the conditions before and after urbanization at the same point (or pixel), provided that the other factors (macroclimate and topographic context) have not changed over time since urbanization began (Table 2.4 on page 39 of Oke et al. [8]). This idea was proposed in the conceptual framework of Lowry [95]. However, an ideal study is most likely impossible because there is typically a lack of records up to pre-urbanization. Thus, to comply with the framework above and to further explore the effect of urbanization on urban climate, in this study, the LSTs of the unchanged urban pixels during the entire study period, $LST\left(u_{0}\right)$, and the difference between the two timestamps were analysed. This urban trajectory method was adopted from the previous studies [[96], [97], [98]].

An unchanged urban pixel ($u_{0}$) is the pixel that is classified as urban in the three timestamps: September 6, 2000 ($t_{1}$), July 29, 2009 ($t_{2}$), and September 11, 2019 ($t_{3}$). This analysis was performed inside the city administrative boundaries of the three selected regions: JMA, BC, and SKB. The rate of the LST change, $\frac{\Delta LST\left(u_{0},t_{m}t_{n}\right)}{\Delta t}$ in °C/year, were calculated by taking the difference in the LST between two timestamps for the same pixel with unchanged urban land cover and divided by the time interval ∆t:

(8) $$\frac{\Delta LST\left(u_{0},t_{m}t_{n}\right)}{\Delta t}=\frac{LST\left(u_{0},t_{m}\right)-LST\left(u_{0},t_{n}\right)}{\Delta t}$$

The three pairs $t_{m}$ and $t_{n}$ are $t_{2}$ and $t_{1}$, $t_{3}$ and $t_{2}$, and $t_{3}$ and $t_{1}$.

#### 2.3.5 Urban thermal field variance index

In this study, the UTFVI was used as an indicator of UHI phenomena [[25], [26], [27], [28], [29],^33^,^99^,^100^]. The UTFVI describes the ecological quality of the urban environment in terms of thermal status by using only one variable, the LST, and is expressed as [25,27,28]:

(9) $$UTFVI\left(i,j\right)=\frac{T_{s}\left(i,j\right)-T_{m}}{T_{m}}$$

where $T_{s}$ is the LST (K) of an individual pixel at location i and j. $T_{m}$ is the area average of the LST (K) inside the city boundary. The UTFVI is one of the scaling techniques. This index can prevent the incomparability of LSTs across timesteps caused by time variation. The thresholds of the UTFVI classes and their interpretations are shown in Table 2. A UTFVI value less than zero indicates the absence of the UHI effect or excellent ecological quality in the area. As the values increase, the intensity of the UHI effect increases, and the thermal conditions or ecological quality degrade from excellent to worst.

**Table 2:** Threshold of the UTFVI and its interpretation.

UTFVI	UHI phenomena	Ecological evaluation index	Class
<0 0–0.005 0.005–0.010 0.010–0.015 0.015–0.020 >0.020	None Weak Middle Strong Stronger Strongest	Excellent Good Normal Bad Worse Worst	L1 L2 L3 L4 L5 L6

#### 2.3.6 Discomfort index

In this study, the DI is adopted for monitoring human thermal comfort conditions via remote sensing. Thom [101] constructed a DI as a measure of the reaction of the human body to a combination of heat and humidity. Thom's equation was a function of dry- and wet-bulb temperatures from ground-based observations. DI can also be calculated by considering temperature and relative humidity [[30], [31], [32], [33], [34],^102^], as in Eq. (10). This formula could be used for nighttime and daytime satellite measurements:

(10) DI=LST−0.55(1−0.01RH)(LST−14.5)

where LST is the land surface temperature in °C and RH is the relative humidity at 2 m in %. RH is derived from t (temperature, °C) and $t_{d}$ (dew-point temperature, °C) at 2 m and approximated by using the equation $RH\approx 100\;-\;5\left(t\;-\;t_{d}\right)$ [103]. The threshold values for the DI categories are shown in Table 3.

**Table 3:** Threshold values of the DI categories [33].

DI temperature (°C)	DI categories	Class
−1.7 to +12.9 +13 to +14.9 +15 to +19.9 +20 to +26.4 +26.5 to +29.9 ≥30	Cold Cool Comfortable Hot Very hot Torrid	D0 D1 D2 D3 D4 D5

#### 2.3.7 Regression analysis

This method is used to examine the relative contributions of different factors influencing SUHII changes. The model was constructed with sample data area-averaged at the subdistrict level inside the urban boundary (delineated base of the 2019 LULC) and combined for the three timesteps for each of the three selected regions. In this study, the contributing factors (independent variables) analysed are the urban ratio (UR), water ratio (WR), vegetation ratio (VR), and anthropogenic heat flux (AH). The ratio is determined by dividing the number of pixels of a particular LULC type by the total number of pixels in a subdistrict. The vegetation pixels are the sum of the LV and DV pixels. The subdistrict level of SUHII (sd-SUHII in °C) is the dependent variable. The sd-SUHII is defined as the LST at each pixel in the urban area minus the average LST area in rural area, which is then averaged at the sub-district level.

Pearson's correlation was conducted prior to regression analysis and showed a high correlation between the independent variables, indicating strong collinearity between the variables. As an alternative, partial least squares (PLS) regression was used to eliminate the collinearity, as in Zhang et al. [50]. PLS creates an orthogonal new set of independent variables (called components) that maximizes the covariance [104]. This technique involves combining the multiple linear regression technique (which finds the best fit between independent and dependent variables) and principal component analysis (which finds a combination of independent variables with large variance and reduced correlation). The optimal number of PLS components was selected by validating the performance of the regression model using the leave-one-out cross-validation [50,105] method by considering the coefficient of determination ($R^{2}$) and mean absolute error.

The resulting equation model is as follows:

(11) $$\hat{\text{sd}‐\text{SUHII}}\left({}^{\circ}C\right)=\alpha_{1}UR+\alpha_{2}WR+\alpha_{3}VR+\alpha_{4}AH+\beta$$

where $\alpha_{i}\left(i=1,2,3,4\right)$ are regression coefficients, β denotes the intercept, and $\hat{\text{sd}‐\text{SUHII}}$ is the expected value of the dependent variable. All independent variables were normalized (subtracted from the mean value and then divided by the standard deviation); therefore, the coefficients (°C) describe the contribution of each variable. A change in one standard deviation of an independent variable i results in an increase (positive) or decrease (negative) in the sd-SUHII by $\alpha_{i}$ °C.

## 3 Results

### 3.1 Land use and land cover dynamics, urbanization, and land surface temperature

**Fig. 2:**
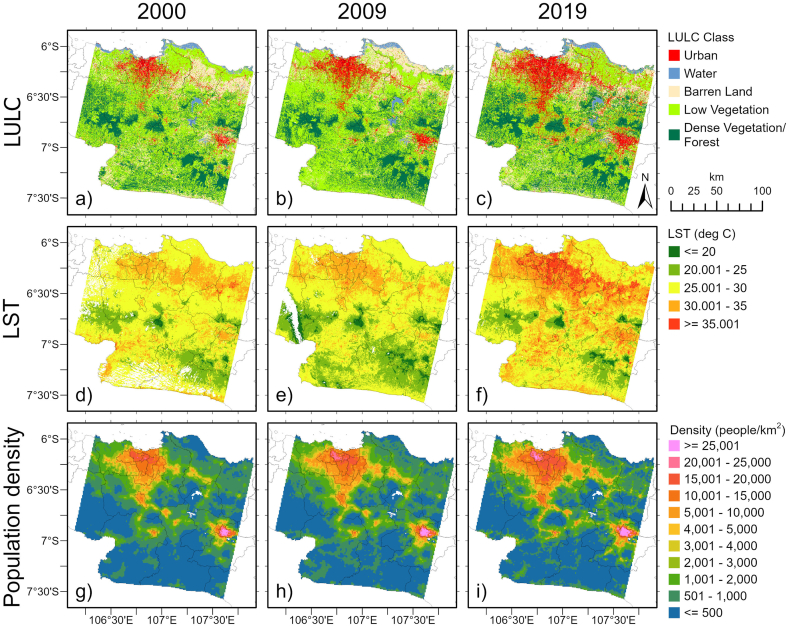
(Top panel) Land use and land cover (LULC) maps in (a) 2000, (b) 2009, and (c) 2019, classified by using the SVM method. (Middle panel) Land surface temperature (LST) maps at instantaneous time (∼10 a.m. LT) on (d) September 6, 2000, (e) July 29, 2009, and (f) September 11, 2019. Areas inside the study area with no color indicate no data due to cloud cover removal. (Bottom panel) Spatial patterns of population density (people/km^2^) across the study area in (g) 2000, (h) 2010, and (i) 2019.

Fig. 2a–c shows the classification results of LULC across the study area in 2000, 2009, and 2019, respectively. The accuracy assessment of the classification is summarized in Table 4. Generally, the classification with SVM provides very good agreement in terms of overall accuracy [79,80] and is acceptable and worthy of interpretation based on the Kappa coefficient [81,82]. For the three years, the overall accuracy and Kappa coefficient are greater than 87 % and 83 %, respectively. For individual classes, the accuracy was calculated by the user's and producer's accuracies. Mostly, those statistical parameters have values higher than 80 %. The accuracy values of some classes are more than 90 % in certain years. One class (low vegetation) has accuracy values lower than 80 % in 2000 and 2019 but still exceeds 75 %.

**Table 4:** Accuracy assessment of LULC classifications for 2000, 2009, and 2019.

Year	Class name	User's accuracy (%)	Producer's accuracy (%)	Overall accuracy (%)	Kappa coefficient (%)
2000	Urban	89.13	94.47	87.05	83.80
	Water	94.20	95.12		
	Barren land	87.76	86.31		
	Low vegetation	75.76	79.55		
	Dense vegetation/forest	89.13	81.35		
2009	Urban	95.98	83.09	88.37	84.86
	Water	98.44	81.82		
	Barren land	81.19	91.62		
	Low vegetation	84.80	92.49		
	Dense vegetation/forest	90.00	87.91		
2019	Urban	93.15	94.67	87.31	83.97
	Water	92.07	96.18		
	Barren land	81.77	89.21		
	Low vegetation	82.14	78.23		
	Dense vegetation/forest	88.45	84.09		

As shown in Fig. 2a–c, the most obvious and extensive urbanization occurred in Jakarta Province and its surrounding areas, known as JMA. Other urbanized areas are also observed in some municipalities, such as Bogor, Sukabumi, Bandung, and Cimahi. Additionally, some urban spots can be found in other small city centers. East of the JMA, the urban pixels formed a line extending from west to east, and they were supposed to follow the road (toll road). Over time, urban pixels developed and extended. The growth of urban areas has been pronounced for the last two decades. The urban area in Jakarta extended to the west, east, and south. Urban pixels were increasingly connected between cities, as observed between Jakarta Province and Bogor Municipality and among other city centers. Moreover, other urban areas, such as those in Sukabumi and Bandung-Cimahi, also developed beyond city administrative boundaries.

Among the other land covers, water pixels had the smallest area (Fig. 2a–c). Large water bodies are observed in the three reservoirs located between BC and JMA. From the south to the north, the reservoirs are the Saguling, Cirata, and Jatiluhur dam reservoirs. Moreover, other water bodies, including ponds and other aquaculture areas, are observed along the northern coast of the study area. For barren land, a fairly large portion of the pixels are located on the north side of the study area, east of the JMA. It is noted from the three maps (Fig. 2a–c) that the LULC types alternate between barren land and low vegetation at these locations. There are extensive paddy fields there [38] where the LULC category can change (either barren land or low vegetation) based on the plantation period, as described in Table 1. Furthermore, barren land pixels might be found in various locations throughout the study area. Moreover, low vegetation composed the majority of the LULC across the study area. Finally, dense vegetation/forest areas are commonly observed on the middle and southern sides of the study area and are found across higher elevations.

Fig. 2d–f shows the LST over the study area at instantaneous time, respectively, on September 6, 2000, July 29, 2009, and September 11, 2019. The color tone used ranges from green (low LST values) to red (high LST values). Areas with no color indicate no data due to cloud cover removal. Qualitatively, the LST in Jakarta Province (above 30 °C) was relatively higher than that in other locations. Over time, the higher LST expanded with increasing magnitude to the west, east, and south of Jakarta Province. Additionally, higher LST values are observed in some municipalities, such as Bogor, SKB, and BC, than in their surroundings. The LST over urban pixels is relatively higher than that over the other LULCs (above 30 °C), while over dense vegetation/forest areas, the LST is the lowest (below 25 °C). The LST of low vegetation areas is approximately 25–30 °C. Moreover, the LST of barren land approaches the LST of urban areas, such as on the east side of the JMA area.

Fig. 2g–i illustrates the population density (people/km^2^) in the 2000, 2009, and 2019, respectively. A higher population concentration is observed in urban areas, such as JMA, Bogor, Sukabumi, Bandung, Cimahi, and various smaller city centers. The rising population density in Jakarta and Bandung is clearly discernible over time and is depicted by a change to pink color in Fig. 2i. In addition, relatively higher densities that extend horizontally are also visible over time, such as in Jakarta, Bogor, and Bandung-Cimahi. A general correspondence existed among the three spatial patterns. Urban areas were expanding, accompanied by increased LSTs and population density.

**Fig. 3:**
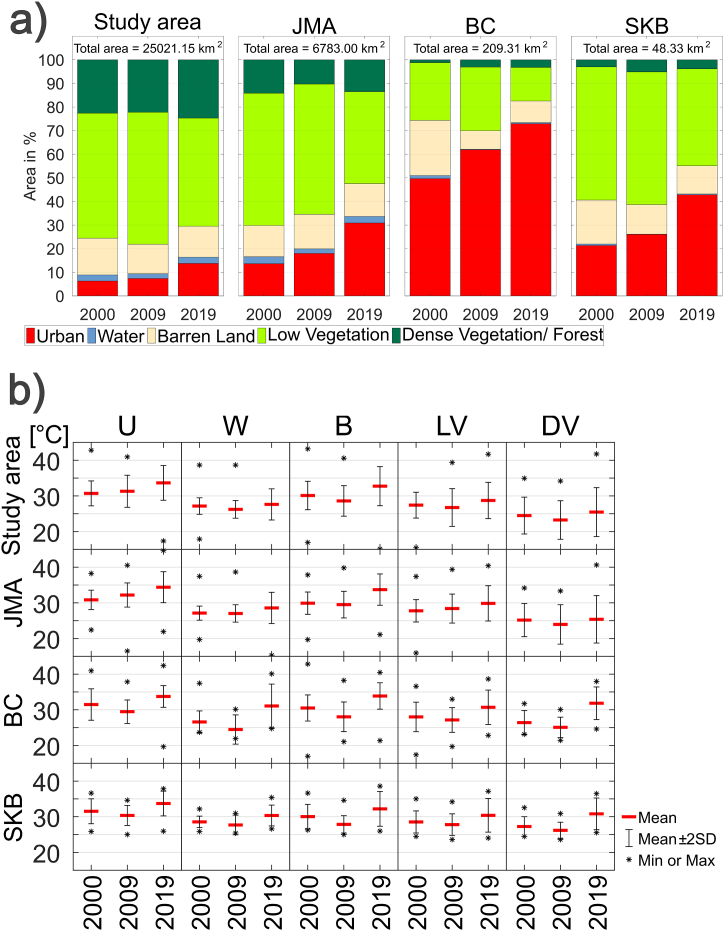
a) The percentage (%) distribution of area for each LULC in the study area (first panel), JMA (second panel), BC (third panel), and SKB (fourth panel). The total area is shown in the graphic in each panel. b) Statistical parameters of LST in the study area, JMA, BC, and SKB for each LULC, namely, urban (U), water (W), barren land (B), low vegetation (LV), and dense vegetation/forest (DV). Only the years are shown on the x-axis, but those are instantaneous for the three timesteps, as shown in Fig. 2d–f.

Fig. 3a shows the percentage distribution (%) for each LULC category in the study area, JMA, BC, and SKB for the three-year period. Over the study area, the number of urban pixels increased from 6.34 % in 2000 to 13.76 % in 2019. The number of water pixels was the lowest in area for the three timesteps, approximately 2 %. The majority of the pixels had low vegetation coverage (52.92 % in 2000), which slightly increased in 2009 but decreased again in 2019. The area of barren land was more than 10 %, and it was fluctuating. Dense vegetation/forest areas accounted for more than 20 % of the total area and slightly increased during the last three timesteps.

Focusing inside the administrative boundary of the JMA, the number of urban pixels increased more than twice during the two decades, from 13.64 % in 2000 to 30.90 % in 2019. It seems that the LULC transition to the urban majority was converted from low vegetation pixels, with the pixels decreasing from 56 % in 2000 to 38.93 % in 2019. Several previous studies [37,39,40,[106], [107], [108]] have also reported that shrinking green space and growing urban areas in Jakarta trigger an increase in the LST and UHI generation. Gandharum et al. [38], whose study focused on the regencies of Bekasi, Karawang, and Cirebon, also reported that the expansion of built-up regions was primarily attributed to agricultural land. The other LULCs might have contributed to the transition to urban areas but in small percentages. Barren land and dense vegetation/forest pixels fluctuated by more than 10 %. The number of water pixels was the lowest, at approximately 2 %.

Within the administrative boundary of BC, urban pixels were the most dominant and have been increasing for two decades, from 49.67 % in 2000 to 72.93 % in 2019. BC is denoted as a saturated city where urbanization extends beyond the administrative city boundary. Firman [59] mentioned that the region grew into a megacity (the Bandung metropolitan area). It appears that pixels of barren land and low vegetation cover were the main sources of transitions to urban regions. Both classes have shown a declining trend for two decades. Moreover, water and dense vegetation/forest occupied the smallest areas in the BC. Similarly, Ramdani and Setiani [109] reported uncontrolled urban expansion and the removal of urban greenery in Bandung, which significantly increased the urban temperature during the study period from 1999 to 2009.

Moreover, in the SKB, the number of urban pixels also increased by more than twofold, from 21.36 % in 2000 to 42.84 % in 2019. Both barren land and low vegetation areas exhibited declining trends. It seems that the extent of urban areas was converted from barren land and low vegetation. In 2019, the percentages of urban and low vegetation areas were comparable. Dense vegetation/forest areas fluctuated between 3 and 5 %. Like in other regions, in this region, the number of water pixels was the lowest, at less than 1 %.

Socioeconomic factors, such as the increase in the urban population resulting from urban migration, are the main drivers behind urban growth in this study area. Moreover, many residents in the middle of a city also disperse into nearby neighbourhoods (where property is less expensive), and the increasing demand for housing forces the horizontal expansion of urban areas. In addition, the demand for land for industrial estates also increased as a result of the swift economic recoveries driven by the profit-based private sectors following the financial crisis in the late 1990s and early 2000s [59]. Infrastructure improvements such as toll roads [107] and shopping centers encourage people to visit cities, which in turn stimulates the growth of the city's economic activities, necessitating land conversion to built-up areas. Deficiencies in the land permission system are another argument in favor of land conversion [59].

Fig. 3b shows some statistical parameters, such as the mean, minimum, and maximum values of LST in the study area and JMA, BC, and SKB for each LULC. By assuming that the LST values follow a normal distribution, approximately 95 % of the values fall within two standard deviations of the mean (in Fig. 3b, the range is mean±2SD). Moreover, any value that falls far outside of two standard deviations of the mean is a possibility as an outlier (some values are outside the vertical axis range of the plotting frame).

In comparison to those of other LULCs, the mean LST values over urban (dense vegetation) areas typically have greater (lower) values (Fig. 3b). In the study area, the mean values of LST over each LULC from the highest to the lowest are urban, barren land, low vegetation, water, and dense vegetation pixels for each year. This result is consistent with previous studies in different locations, such as Italy [25], Bangladesh [26], Brazil [34], and India [29]. The highest LST values are found in built-up and barren areas because those LULC types absorb more heat. Moreover, urban regions also have extensive concrete construction, which radiates much more heat and warms the surface, increasing the LST. Water bodies and vegetation cover present lower LST values due to evaporation, shading processes, heat absorption, and heat loss through transpiration [25,26,29,34]. The presence of soil and plant water moisture provides mechanisms for heat loss by latent heat fluxes and evaporative cooling [8].

In the urban pixels of the study area, the average LST at one time was greater than that in the previous time frame (Fig. 3b). Moreover, in the other LULCs, the average LST value in the middle year (2009) was lower than that in the previous year (2000). Similar patterns were also found in JMA. The mean values of LST over urban pixels have the highest values each year. In BC and SKB, in general, the mean LST values over urban areas were greater than those over the other LULC categories, except for BC in 2019, where the mean LST over barren land was slightly greater than the mean LST over urban pixels. For all LULC categories, the mean LST values in the middle year (2009) were lower than those in 2000. The spatial pattern of the LST (Fig. 2d–f) also shows that generally, in BC and SKB, the mean LST in 2009 was lower than that in 2000, but this difference was not observed in JMA. Feng et al. [110] mentioned that certain anomalous values could be found by using several images because they could not depict full-time variations. There is likely an anomaly in the macroclimate that affects the LST, which will be discussed in subsection 4.2.

### 3.2 Effects of urbanization on land surface temperature

#### 3.2.1 Spatiotemporal variations in the surface urban heat island

**Fig. 4:**
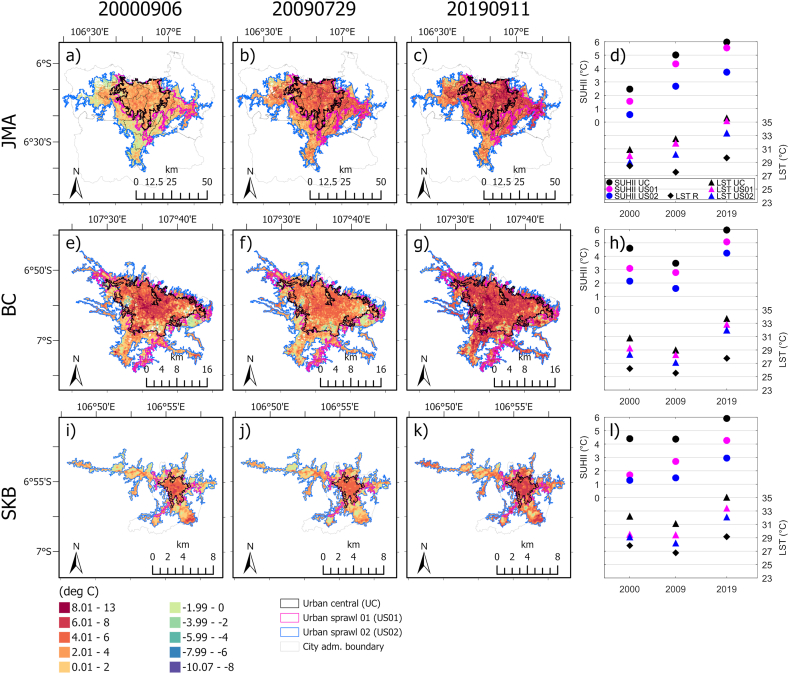
(The first three column) spatial patterns of LST over each pixel in the urban areas (UC, US01, and US02) minus the area average of LST in the rural zone (R) in JMA (first row), BC (second row), and SKB (third row) on September 6, 2000 (first column), July 29, 2009 (second column), and September 11, 2019 (third column). (The last column) three types of SUHII and area average LST for the three urban areas and a rural area in JMA, BC, and SKB at the three timesteps. Only the years are shown on the x-axis, but those are instantaneous for the three timesteps.

The first three columns of Fig. 4 show the spatial pattern of LST deviation within the three defined urban boundaries (UC, US01, and US02) with respect to the rural area in JMA, BC, and SKB. The UC, US01, and US02 boundaries were clustered urban in 2000, 2009, and 2019, respectively, which also reveals the rate of urban expansion in all three areas. In general, the urban areas are growing beyond the administrative boundaries, with outstanding expansion over the last two decades. In JMA, the UC (black line) in 2000 was formed in Jakarta Province. In 2009, the urban sprawl (magenta line) expanded to the south and east. Then, in 2019, it expanded further to the west, south, and east sides and became one cluster merged with Bogor Municipality (blue line). In BC, the UC had formed beyond the administrative boundaries by 2000. In 2009 and 2019, urban sprawl expanded to the west, south, and east and grew into the Bandung and West Bandung (Bandung Barat) regencies. However, the urban growth on the north side is limited by mountainous areas and conservation forest areas. In the SKB, the UC in 2000 was in the middle of the administrative boundary. In the following decade, urban sprawl grew but was not too intensive. However, in the last decade, urban areas have developed rapidly beyond administrative boundaries, such that extensive growth has occurred on the western side in line form. It seems that the line form follows the road orientation.

Mostly, the LST in urban areas is relatively higher than that in rural areas (positive deviations), as shown by the yellow to red colors. Urban areas experience greater surface temperatures than rural areas due to a combination of variables [8], namely, 1) radiative and thermal properties, including albedo and emissivity for the former property and heat capacity, thermal conductivity, diffusivity, and admittance for the latter; 2) the availability of moisture for evaporative cooling, while urban soil moisture is probably lower; and 3) urban structure: the complex 3D shape of the city promotes increased energy exchange, and canyon trapping results in numerous shortwave radiation reflections and a reduction in longwave radiation losses.

However, some pixels in the urban zones had lower LSTs than did those in the rural area (negative values, shown by green to blue colors), which can be observed mostly in the US01 and US02 in the first timestep (September 6, 2000) and in the US02 in the second timestep (July 29, 2009). During those times, the zones have fewer urban pixels and more greenery (vegetation). While inside the UC, greenery (vegetation) and water bodies might affect the lowering of LST. This is also supported by Fig. 3b, which shows that water and vegetation pixels generally have lower LSTs inside the cities. As mentioned by previous studies [27,99,100,111], this study also suggested that vegetation cover has an important role in alleviating urban heat temperatures and supporting the sustainable development of cities.

The last column of Fig. 4 shows the daytime SUHIIs (filled circle symbols) in JMA, BC, and SKB for the corresponding SUHII UC (black circles), SUHII US01 (magenta circles), and SUHII US02 (blue circles). The highest to lowest SUHIIs are SUHII UC, SUHII US01, and SUHII US02, respectively. It shows that the SUHII in the urban centers is higher than the SUHII in urban sprawls. The three types of SUHIIs in JMA increases over two decades from 2.46 to 5.97 °C (for SUHII UC), from 1.55 to 5.54 °C (SUHII US01), and 0.56–3.73 °C (SUHII US02). In BC, the SUHIIs ranged from 3.47 to 5.96 °C (for SUHII UC), 2.78–5.07 °C (for SUHII US01), and 1.59–4.23 °C (for SUHII US02). In SKB, the SUHII UC fluctuated between 4.37 and 5.91 °C. While SUHII US01 and SUHII US02 were both increasing from 1.69 to 4.27 °C and 1.29–2.95 °C, respectively. Siswanto et al. [47] reported SUHII of approximately 3–6 °C in JMA. Those values are comparable with this study in SUHII UC of JMA. However, a small difference may be due to differences in calculations. They calculated the SUHII based only on the difference in LST between two points: urban (Kemayoran) and rural (Citeko) areas. Moreover, a positive trend in the SUHII was reported in Bogor [46] as consistent with an increasing pattern of SUHII in this study, except in the middle timestep of SUHIIs in BC and SUHII UC in SKB.

The mean LSTs over urban zones (filled triangles) and a rural zone (filled diamonds) are also plotted. For the three cities, the mean LSTs in UC are the greatest, followed by LSTs in US01, US02, and rural zones, respectively. In JMA, the mean LSTs of the urbans (triangles) exhibited an increasing pattern for the three timesteps. While the mean LST of rural (diamond) fluctuated and got the lowest in the middle timestep. In BC and SKB, the mean LSTs in 2009 for urban and rural areas were lower than those in 2000 and 2019, following the same pattern as that shown in Fig. 3b.

#### 3.2.2 Temperature variation of the unchanged urban pixels

**Fig. 5:**
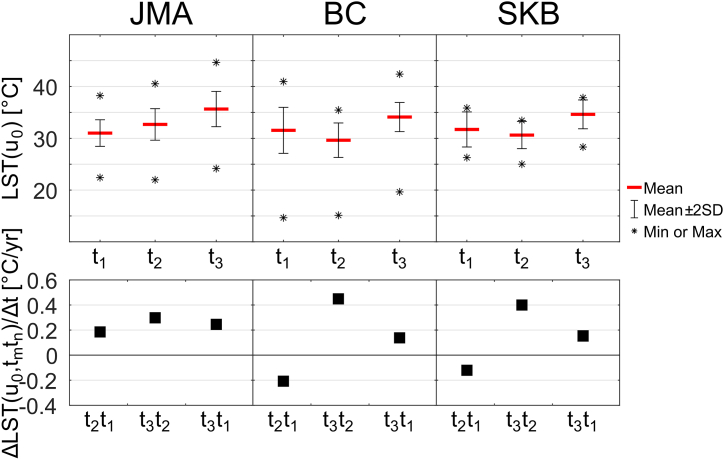
(Top panel) Statistical parameters of LST of the unchanged urban pixels, $LST\left(u_{0}\right)$, on September 6, 2000 ($t_{1}$), July 29, 2009 ($t_{2}$), and September 11, 2019 ($t_{3}$) in JMA, BC, and SKB. (Bottom panel) Area average of the difference in the LST of the unchanged urban pixel between two timestamps divided by time interval, $\Delta LST\left(u_{0},t_{m}t_{n}\right)/\Delta t$; see Eq. (8). All values are statistically significant (Student's *t*-test) at *p* < 0.01.

Fig. 5 shows the temporal variation in the LST of the unchanged urban pixels inside the administrative boundaries of JMA, BC, and SKB. The top panel shows the statistical parameters (mean, minimum, maximum values, and distribution) of the LST over the unchanged urban pixels. The mean values of BC and SKB during the middle time were lower than those during the first and third times, which was consistent with the previous figures (Fig. 3, Fig. 4h and 4l). A temporal pattern similar to that of the mean value is generally also shown by the maximum and minimum values. In JMA, the daytime LST of the unchanged urban pixels ranged from 22.41 to 38.24 °C, 21.98–40.53 °C, and 24.16–44.65 °C for 2000, 2009, and 2019, respectively. Meanwhile, in BC, the ranges were from 14.65 to 40.95, 15.12 to 35.45, and 19.64 to 42.40 °C for consecutive of the three timesteps. Smaller ranges of the LST were observed in SKB, with magnitudes ranging from 26.27 to 35.84, 25.00 to 33.43, and 28.32 to 37.84 °C in the three timesteps, respectively.

The bottom panel of Fig. 5 shows the difference in the mean LST between two time points divided by the time interval (rate of the LST change). In the x-axis, the symbol $t_{2}t_{1}$ represents the mean LST value at the second time point minus the value at the first time point. The two other symbols have similar interpretations. In JMA, the rate of the LST change between the two sampling times always increased, from approximately 0.19 °C/yr in the first decade ($t_{2}t_{1}$) to 0.30 °C/yr in the second decade ($t_{3}t_{2}$). However, the rate within a period of two decades ($t_{3}t_{1}$) is 0.25 °C/yr. In BC and SKB, the patterns of the rate are similar, with negative values in the first decade of approximately −0.21 and −0.12 °C/yr, respectively. In the second decade, the rates are 0.45 °C/yr in BC and 0.40 °C/yr in SKB. For almost two decades, the rate values are 0.14 and 0.15 °C/yr for BC and SKB, respectively.

#### 3.2.3 Variations in urban thermal conditions

**Fig. 6:**
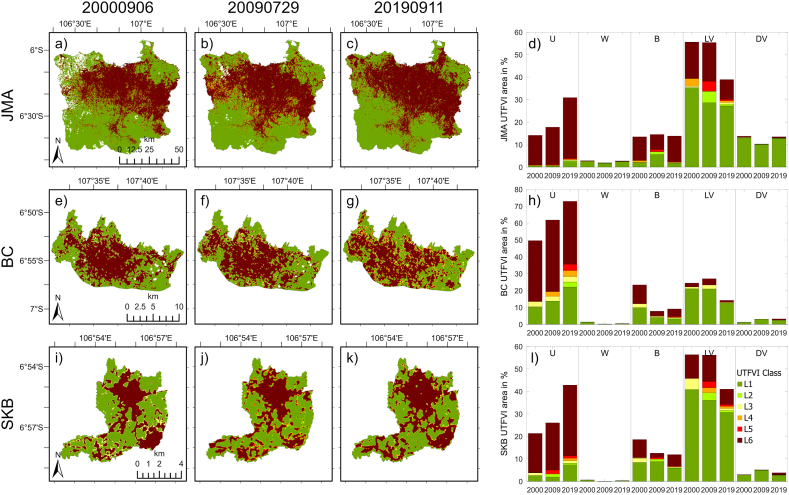
(First to third columns) Spatial patterns of the UTFVI in JMA (first row), BC (second row), and SKB (third row) on September 6, 2000 (first column), July 29, 2009 (second column), and September 11, 2019 (third column). (Fourth column) Percentage (%) area of UTFVI categories in different LULC classes in JMA (first row), BC (second row), and SKB (third row). Only the years are shown on the x-axis, but those are instantaneous for the three timesteps.

Fig. 6 shows the spatial pattern of the UTFVI (the leftmost three columns of plots) and its distribution within LULC types (the rightmost column) in JMA, BC, and SKB at the three time points. The pixelwise values of the indices were categorized into six classes, from L1 (no UHI phenomena and excellent ecological quality) to L6 (the strongest UHI phenomena and the worst ecological evaluation), as shown in Table 2. From the spatial maps, L6 (dark red) and L1 (dark green) are the most common classes inside the three regions. A similar spatial pattern was also shown in previous results [27,28,99]. Over time, the class L6 became wider in JMA and SKB. Moreover, in the JMA, L6 extended primarily southwards to the Bogor Municipality. In BC, the spatial expansion of L6 was less obvious.

According to the rightmost column of Fig. 6, the L6 category dominates in urban pixels throughout the three regions. Moreover, L1 represents in most of the LV pixels. These results are also consistent with previous studies [25,26,29]. Moreover, these results suggest that protecting and increasing vegetation coverage can mitigate the adverse effects of high urban temperatures, as reported by Zhang et al. [100].

In the urban (U) pixels of JMA (Fig. 6d), areas in the L6 category were increasing over time, with distributions of 13.28 %, 16.65 %, and 27.03 % for 2000, 2009, and 2019, respectively. Moreover, the percentage area of the other classes was less than 1 %, except for the L1 category, which was approximately 2 % in 2019. In addition, the number of water (W) pixels was very small, with the majority in the L1 category in the three timesteps. Moreover, the total area of barren land (B) was more than 10 %, with the majority of UTFVI categories being L6. In 2009, the L1 category increased by less than half of the barren land area. On the other hand, the number of low vegetation (LV) pixels was the largest, with the majority occurring in the L1 class. However, it is intriguing that the L6 category had the second-largest proportion of LVs among the three timesteps. Finally, for dense vegetation/forest (DV), the L1 category was the most dominant for the three timesteps.

In the BC (Fig. 6h), the L6 category accounted for more than half (majority) of the urban pixels, and the second largest proportion was L1. In addition, the remaining categories of the UTFVI constituted a minor proportion of the pixels. Over time, the proportions of *strong* to *strongest* UHI phenomena (categories L4 to L6) are 36.18 %, 45.30 %, and 44.62 %, respectively. In the water pixels, even though the area was very small, the L1 category was dominant. On the other hand, the percentages of L1 and L6 in barren land were comparable, except in 2009, when the L1 category was larger in proportion than L6. Moreover, most LV pixels were classified as L1, and L6 was also present, but in a small proportion. Moreover, in the DV, the majority category was L1. In the last region (Fig. 6l), SKB, most urban pixels were in category L6. Through time, the proportions of categories L4 to L6 were increasing from 17.58 %, 22.77 %, and 33.64 % for the three timesteps, respectively. In addition, the water percentage was very small and dominated by the L1 category. Like those of BC, the percentages of L1 and L6 in barren land were comparable, except in 2009. While the L1 category dominated the LV and DV pixels, L6 was also available in both types of LULC.

#### 3.2.4 Variations in thermal comfort

**Fig. 7:**
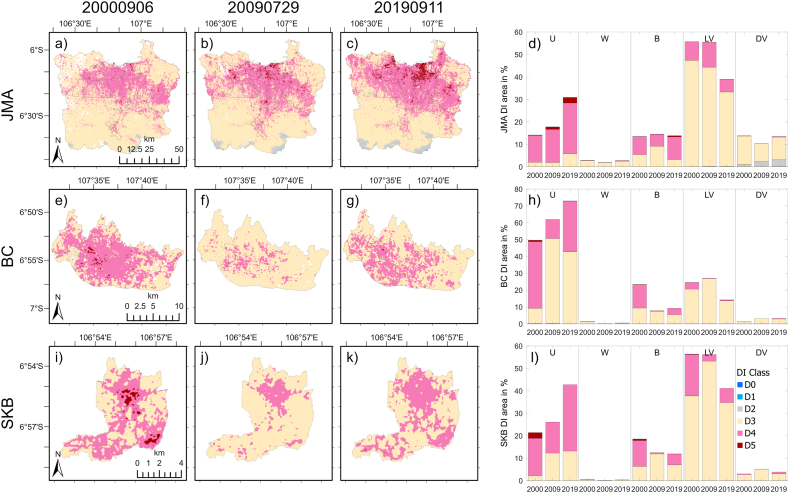
(First to third columns) Spatial patterns of DI in JMA (first row), BC (second row), and SKB (third row) on September 6, 2000 (first column), July 29, 2009 (second column), and September 11, 2019 (third column). (Fourth column) Percentage (%) area of DI categories in different LULC classes in JMA (first row), BC (second row), and SKB (third row). Only the years are shown on the x-axis, but those are instantaneous for the three timesteps.

Fig. 7 shows the spatial patterns of the DI (the leftmost three columns of plots) and its distribution for each LULC type (the rightmost column) in JMA, BC, and SKB at the three time points. The pixelwise value of the index was categorized into six classes, as shown in Table 3, from D0 to D5. From the spatial patterns, most proportions are in D4 (pink) and D3 (yellow) inside the three regions. D4 and D3 are *very hot* and *hot* categories, respectively. Generally, as shown in the figure, the majority class found in urban areas is D4. Several pixels of the D5 (torrid) class appeared in the middle of the city center, as shown in Fig. 7c–e, and i. According to the three timestamps, the D4 class became wider in the JMA. In addition, the extents of D4 were mostly to the south. Moreover, in BC and SKB, the area of the class in the second column was smaller. This difference might be due to the anomalous pattern of the LST during that time.

The rightmost column of Fig. 7 shows the percentage (%) area of DI classes for each LULC in JMA, BC, and SKB. In urban pixels, the D4 category was dominant in JMA and SKB for the three timestamps and in BC for the first timestamp (2000). Moreover, for the other two time periods (2009 and 2019), D3 dominated the urban pixels in the BC.

In JMA (Fig. 7d), the D4 class area in urban (U) pixels was more than 10 % for 2000 and 2009 and more than 20 % for 2019. Moreover, areas of the other classes were less than 1 %, except for the D3 category for the three timesteps and D5 in 2009 and 2019. In addition, the number of water (W) pixels was very small, with the majority in the D3 category. For barren land (B), D3 (D4) dominated in 2000 and 2019 (2009). Moreover, most of the LV pixels were in D3 for the three timestamps. Moreover, D4 has the second-largest proportion. Finally, D3 was the most dominant class over DV, but D2, which is also the comfortable class, was also the second most common class.

In the urban pixels of BC (Fig. 7h), D4 (D3) was dominant in 2000 (2009 and 2019). Moreover, the other classes of DI were minor in urban areas. Moreover, in water pixels, D3 was dominant. For barren land, D4 (D3) was dominant in 2000 (2009 and 2019). For LV and DV, the D3 class dominated. In the SKB (Fig. 7l), most urban pixels were in category D4, and D5 was also observed in 2000. The D3 category was the second largest in urban pixels. Moreover, the number of water pixels was small, and the D3 category dominated. Moreover, D3 (D4) was the dominant variable in barren land pixels in 2009 and 2019 (2000). Finally, the D3 class dominated in the LV and DV, but D4 was also available in those pixels.

### 3.3 Contributing factors to surface urban heat island variations

**Fig. 8:**
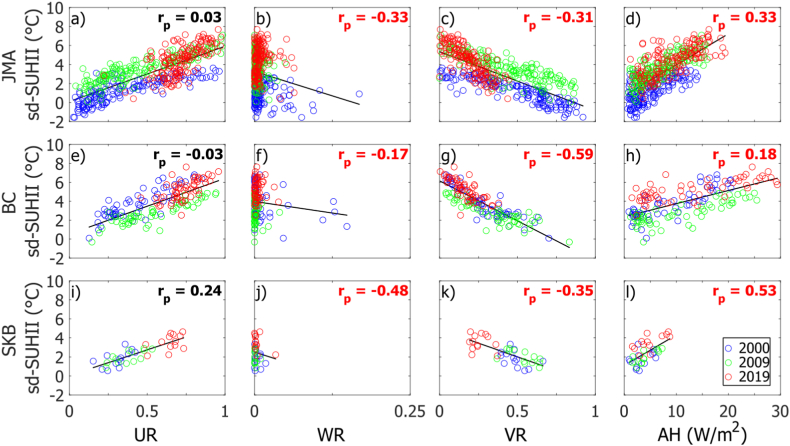
Scatter plots showing the relationships between the subdistrict level of SUHII (sd-SUHII, °C) and urban ratio (UR), water ratio (WR), vegetation ratio (VR), and anthropogenic heat flux (AH, ${W/m}^{2}$) for JMA (first row), BC (second row), and SKB (third row). The samples used are the averages from the subdistrict administration for the three years. The colors in the circles indicate data from different years. Partial correlations ($r_{p}$) are shown at the right top of each subplot, with significant value in red. The linear regression (black line) and partial correlation were calculated by combining samples from the three years.

Fig. 8 shows scatter plots between the subdistrict level of SUHII (sd-SUHII) and each variable that influences changes in the SUHII, such as UR, WR, VR, and AH, for the three urban areas. It is noted that the sd-SUHII extracted only at instantaneous time around 10 a.m. LT. The black line in each plot estimates linear regression between two variables. From the regression lines, it is shown that UR and AH have a positive relationship with sd-SUHII, while WR and VR have a negative relationship with sd-SUHII. The partial correlation was also performed to show how strong the relationship between two variables was while suppressing the effects of the other variables. The correlation value ($r_{p}$) is shown at the top right of each plot. Mostly, the sign of either positive or negative values is consistent with the regression, except for the relation between UR and sd-SUHII in BC (Fig. 8e), which has a negative value. This unsignificant negative relationship is difficult to interpret. However, the low and unsignificant partial correlation between UR and sd-SUHIII in the three cities might be due to the strong relationship between UR and the other factors such as VR and AH. However, generally, the regression results of this study are consistent with previous studies [[112], [113], [114], [115]].

The relative contribution of each of the factors above was quantified through the PLS regression equation shown in Table 5. For the three urban areas, the coefficients for UR and AH are positive, meaning that increasing these variables leads to an increasing SUHII. On the other hand, the coefficients for WR and VR are negative; increasing WR or VR leads to a decreasing SUHII. These results are also consistent with the previous results [49,50]. Interestingly, for JMA and BC, the contribution of AH is significantly higher than that of UR in increasing SUHII. Meanwhile, in the SKB, the contribution of UR is slightly higher than that of AH. Moreover, adding vegetation has the highest contribution to reducing SUHII in JMA and BC, as indicated by the largest contribution of VR.

**Table 5:** Statistical metrics (coefficient of determination ($R^{2}$), correlation (r), and p value) calculated from the PLS regression analysis between the fitted and observed sd-SUHII.

Area	Fitted regression equations	$R^{2}$	r	p
JMA	$\hat{\text{sd}‐\text{SUHII}}\left({}^{\circ}C\right)=0.21\;UR-0.37\;WR-0.94\;VR+0.58\;AH+3.07$	0.71	0.84	0.00
BC	$\hat{\text{sd}‐\text{SUHII}}\left({}^{\circ}C\right)=0.09\;UR-0.19\;WR-1.21\;VR+0.15\;AH+3.90$	0.75	0.87	0.00
SKB	$\hat{\text{sd}‐\text{SUHII}}\left({}^{\circ}C\right)=0.43\;UR-0.28\;WR-0.36\;VR+0.34\;AH+2.43$	0.80	0.90	0.00

## 4 Discussion

### 4.1 Implications for urban planning

Studies on global cities [94,116] have highlighted Indonesia's leading position in terms of urbanization's impact on increasing SUHII (as depicted in Fig. 6 of Li et al. [94]) and its significant contribution to regional and global surface warming (referenced in Table 1 of Zhou et al. [116]). Therefore, further analysis of urbanization in Indonesian contexts holds significant value for local urban management and planning, facilitating the identification of specific heat concentration locations within cities and prioritizing areas for urban heat prevention measures.

While these global studies offer insights into spatial variations across cities worldwide, they often lack detailed spatial information within individual cities. Hence, expanding the analysis within each city becomes imperative to obtain a more nuanced spatial understanding. For instance, a study by Rizvi et al. [117] in Karachi demonstrated that as urbanization intensifies, SUHII strengthens and the SUHI area expands (illustrated in Figs. 2 and 6 of their study). Similarly, our investigation of three Indonesian cities underscores the escalating strength of SUHII over time due to rapid urban expansion (as depicted in Fig. 4 in this study). Additionally, more detailed variations of SUHII within the cities and the direction of urban growth for the three cities are well represented (as showcased in the spatial plots in Fig. 4).

Although in general SUHII in urban centers is higher than SUHII in urban sprawls, several hotspot points with elevated heat concentrations can be observed in all three city cases, including some clustered areas in the urban center and western and eastern parts of the urban sprawl in JMA, various zones within the urban centers of BC and SKB, and several locations in their urban sprawls. These detailed spatial variations in SUHII within cities offer valuable insights for urban planners to identify specific areas with elevated SUHII concentrations and prioritize locations for implementing SUHI mitigation strategies, such as allocating green spaces or implementing other countermeasures. Moreover, we strongly advocate conducting similar detailed analyses across more cities in the Indonesian region in future studies.

The contributing factors analysed in this study provides insight into unique action options for each city in terms of the population for urban heat island mitigation that can be adopted in urban planning. As mentioned in the previous section, for JMA and BC, a reduction in AH has a greater contribution than UR to reducing the SUHI. Thus, for higher population density cities such as JMA and BC, the government could limit and reduce population density or encourage energy-efficient and low-carbon building design to reduce anthropogenic heat flux. In contrast, in SKB, a city with a lower population density, limiting urbanization and reducing anthropogenic heat has a more comparable contribution to reducing the SUHII. In addition, for all cities, it would also be possible to introduce additional greenery and water bodies even in the existing urban centers, as well as by regulations, as lessons learned from the Cheonggye stream in the middle of Seoul, which can reduce the temperature of the local environment [118,119].

Currently, Indonesia will move its capital city from Jakarta to East Kalimantan, and this change is expected to reduce urban heat in Jakarta. However, this expectation might be overly optimistic. Since Jakarta is also the economic center of the country, it is likely to have a large influx of workers each year who will live either in the city center or in the suburbs. Consequently, urbanization will likely continuously expand vertically and horizontally. On the other hand, the development of a mega-urban connecting Jakarta and Bandung [59] will also continue and will have an impact on increasing horizontal urbanization in the future, especially with the presence of high-speed railways connecting the two cities.

Based on the patterns of urban growth observed over the last two decades and some reasoning presented in the previous paragraph, the possibility of continued urban expansion in the study area is expected to increase. Urban expansion without the restoration process of green infrastructure and ecological conditions has a local impact on worsening urban warming and thermal conditions, as reported by previous studies [120,121] with study areas in several cities in China, which will also be further exacerbated by the resultant effect of global warming, as shown in the case of Hanoi, Vietnam, by Kubota et al. [122] and Lee et al. [12]. It is important for the government to carefully determine action planning and policy implementation on a scientific basis.

### 4.2 Limitations of this study and suggestions for future research

This research has several limitations. the LST datasets are snapshots at a specific time, and it is difficult to represent a yearly average with only one snapshot. By analysing the three scenes with instantaneous observations for the past two decades, it also might suffer from the high uncertainty of inter-annual trends. However, to maximize the representativeness, images with minimum cloud cover within the same season (during the dry season) were chosen, and no rainy days were observed around the selected days in the study area. Through these treatments, we assume that, in the context of urbanization effects, the interannual comparisons are hopefully more comparable. However, for future studies, it will be valuable to use more samples and denser time-sampling of satellite images.

LST variation is affected not only by urban growth but also by non-urbanization factors, such as local and macroclimate signatures, which are also included in the dataset; therefore, assumptions that their effects may be minimized must be addressed. For JMA, which is a coastal city, the LST could be influenced by the sea breeze (local climate). For the SUHII analysis, it might be assumed that both urban and rural areas receive similar wind effects from the local climate, so the difference in LST between the urban and rural areas could eliminate the local climate contribution. For the LST analysis of the unchanged urban pixels, the local effect might also be ignored when calculating the LST difference (Eq. (8)) by using data at the same time (10 a.m.) with the assumption that the local climate is similar at all three timestamps.

On the other hand, macroclimate conditions, such as the El Niño-Southern Oscillation (ENSO), also influence LST variations. During El Niño (the warm phase of ENSO), the sea surface temperature in the Indonesian territory is lower than normal [125]. Note that the satellites’ images for LST calculation were at instantaneous time in September 2000, July 2009, and September 2019. During those months, the Ocean Niño Index (ONI) has values of −0.5 (La Niña), +0.5 (El Niño), and 0.2 (Normal), respectively. The ONI is available at the website of the Climate Prediction Center of NOAA (https://origin.cpc.ncep.noaa.gov/products/analysis_monitoring/ensostuff/ONI_v5.php).

Therefore, the LST datasets from the three timesteps are affected by different macroclimate regimes. As shown in Fig. 3, Fig. 4h and 4l, and 5, the LST in 2009 was lower than that in 2000. As El Niño occurred in September 2009, El Niño could influence the anomalous pattern in 2009. However, the results of this study differ from those of previous studies, which showed that the LSTs in Kalimantan (Indonesia) [123] and Kuching city (Malaysia) [124] had higher values during El Niño. Additional analysis is provided in the SI. For the SUHII analysis, it might also be assumed that both urban and rural areas receive the same macroclimate effect, so the macroclimate contribution could be eliminated during the LST difference calculation. This study suggested that caution should be exercised in interpreting the LST patterns of urban areas because LST variations are affected not only by urban growth but also by other factors (such as local and global climates) that contribute to the LST signal.

Another limitation is the small number of city samples in the regression analysis, which were only three cities. It affected the limited diversity in terms of population density in the sampled cities. With a larger sample size, more general results will be obtained regarding the uniqueness of urban heat mitigation actions for cities with larger ranges of population densities. In future research, additional samples should be analysed and collected from various cities in Indonesia using data with higher spatial and temporal resolutions.

## 5 Conclusion

This comprehensive study classified the LULC and examined urban expansions over the last two decades in the western part of Java Island, Indonesia, and three urban areas (JMA, BC, and SKB). The effects of urbanization on surface temperature and its relationship with urban growth were also analysed via remote sensing, as were the contributing factors to SUHI intensity. The main dataset used in this study is Landsat images from 2000, 2009, and 2019. The SVM approach was used to classify LULC. The LST was calculated from Landsat images with the scene center time at approximately 10 a.m. local time. The SUHII was quantified by calculating the temperature difference between urban and rural areas, and the city clustering algorithm was used to cluster the urban area. Three types of daytime SUHII were quantified, namely SUHII urban central (UC) and two SUHII urban sprawls (US01 and US02). In addition, an analysis of the LST variations in unchanged urban pixels over the last two decades was performed. Two indices, the UTFVI and DI, were also analysed to describe the UHI phenomenon, the ecological quality of the urban environment, and urban thermal comfort.

The main findings of this study are as follows.

• The classification results obtained with the SVM model exhibit very good agreement in terms of overall accuracy and are acceptable according to the Kappa coefficient. The overall accuracy and Kappa coefficient are greater than 87 % and 83 %, respectively.
• In the last two decades, urbanization has increased significantly in the study area. Within the JMA and SKB, urban pixels have more than doubled in the last two decades. Some urban areas, such as those in BC and SKB, developed beyond administrative boundaries.
• The SUHII urban central area is greater than the SUHII urban sprawl areas in all three cities. Over the two decades, the SUHIIs in JMA rose, with SUHII UC rising from 2.46 to 5.97 °C, SUHII US01 from 1.55 to 5.54 °C, and SUHII US02 from 0.56 to 3.73 °C. In comparison, the SUHIIs of metropolitan cities in several Asian developing countries are more than 4 °C for Bangkok (Thailand) [13,18] and 2.74 °C for Dhaka (Bangladesh) [15], and in the range of 10.5–14 °C for Indian cities [14]. SUHII UC, SUHII US01, and SUHII US02 in BC varied from 3.47 to 5.96 °C, 2.78–5.07 °C, and 1.59–4.23 °C, respectively. In SKB, the SUHII UC varied from 4.37 to 5.91 °C, while the SUHIIs US01 and US02, respectively, increased from 1.69 to 4.27 °C and 1.29–2.95 °C.
• For the unchanged urban pixels during the last two decades, the three cities showed rate of the LST change (°C/year) with magnitudes of 0.25, 0.15, and 0.14 for JMA, SKB, and BC, respectively.
• For the three regions, *the strongest UHI phenomenon and the worst ecological evaluation* category of UTFVI is the most prevalent in urban pixels, while *no UHI phenomenon* category is the most common in vegetated pixels. According to the DI results, inside the three regions, the majority proportions are in *very hot* and *hot* categories.
• In all three urban areas, the increase in urban ratio and anthropogenic heat flux contributed to the increase in SUHII. On the other hand, the increase in vegetation ratio and water ratio contributed to the decrease in SUHII. These factors have different relative contribution magnitudes to SUHII variations in the three cities.

The government or stakeholders will also find this study useful, as it represents recent urbanization and its impacts (e.g., urban heat) in the study area and could be used for sustainable city evaluation. The forest city concept (characterized by a predominance of forest and tree cover) that will be implemented to develop Indonesia's new capital city, Nusantara, might mitigate the adverse effects of urban heat. However, to prevent uncontrolled urban sprawl in the future, the rules must be strictly enforced and administered to the surrounding cities as well. More studies for the new capital city should be conducted carefully, including comprehensive scientific research on LULC changes and their impacts on the urban microclimate using advanced downscaling methods.

## Data availability statement

Data will be made available on request.

## CRediT authorship contribution statement

**Faiz Rohman Fajary:** Writing – original draft, Visualization, Validation, Software, Resources, Methodology, Investigation, Formal analysis, Data curation, Conceptualization. **Han Soo Lee:** Writing – review & editing, Supervision, Project administration, Investigation, Funding acquisition, Formal analysis, Conceptualization. **Tetsu Kubota:** Writing – review & editing, Supervision, Investigation, Funding acquisition, Formal analysis. **Vinayak Bhanage:** Writing – review & editing, Methodology, Investigation, Formal analysis. **Radyan Putra Pradana:** Writing – review & editing, Investigation, Formal analysis. **Hideyo Nimiya:** Writing – review & editing, Supervision, Formal analysis. **I Dewa Gede Arya Putra:** Writing – review & editing, Formal analysis, Data curation.

## Declaration of competing interest

The authors declare that they have no known competing financial interests or personal relationships that could have appeared to influence the work reported in this paper.

## Figures and Tables

**Table A.1:** Information about the Landsat images used in this study. The dates in bold text show the dates of the images with the least cloud cover. The scene center times shown are only for the images with bold dates.

Year	Landsat	Path/Row	Date	Scene center time
2000	5 TM C2 L2 (L1)	122064	**20000906**; 20010808	02:38:12.3360630Z
		122065	**20000906**; 20000720	02:38:36.4700130Z
2009	5 TM C2 L2 (L1)	122064	**20090729**; 20090830	02:49:08.9720940Z
		122065	**20090729**; 20090713; 20090830	02:49:33.0880940Z
2019	8 OLI/TIRS C2 L2 (L1)	122064	**20190911**; 20190810	03:00:20.1952990Z
		122065	**20190911**; 20190810	03:00:44.1202270Z
