# Supplementary material for: Comprehensive spatiotemporal evaluation of urban growth, surface urban heat island, and urban thermal conditions on Java island of Indonesia and implications for urban planning
Source: Heliyon. 2024 Jun 27;10(13):e33708. doi: 10.1016/j.heliyon.2024.e33708 (PMC11269843; doi:10.1016/j.heliyon.2024.e33708)
Supplement: Multimedia component 1 [file mmc1.docx]

**Supplementary Information (SI)**

***for***

**Comprehensive spatiotemporal evaluation of urban growth, surface urban heat island, and urban thermal conditions on Java Island of Indonesia and implications for urban planning**

**Faiz Rohman Fajary^a,b,^**^[[1]](#footnote-1)^***, Han Soo Lee^a,c,^**^[[2]](#footnote-2)^***, Tetsu Kubota^a^, Vinayak Bhanage^a^, Radyan Putra Pradana^a,e^, Hideyo Nimiya^d^, I Dewa Gede Arya Putra^d,e^**

1. Transdisciplinary Science and Engineering Program, Graduate School of Advanced Science and Engineering, Hiroshima University, 1-5-1 Kagamiyama, Higashi-Hiroshima, 739-8529, Hiroshima, Japan
2. Atmospheric Science Research Group, Faculty of Earth Science and Technology, Institut Teknologi Bandung, Bandung, 40132, West Java, Indonesia
3. Center for Planetary Health and Innovation Science (PHIS), The IDEC Institute, Hiroshima University, 1-5-1 Kagamiyama, Higashi-Hiroshima, 739-8529, Hiroshima, Japan
4. Graduate School of Science and Engineering, Kagoshima University, 1-21-40 Korimoto, 890-0065, Kagoshima, Japan

^e^ Center for Research and Development, Indonesian Agency for Meteorology Climatology and Geophysics (BMKG), Jl. Angkasa 1 No. 2, Kec. Kemayoran, 10610, Jakarta, Indonesia


$=$**SI-A: Average energy consumption**

Average energy consumption per person $\left[ E_{avg} \left( W/person \right) \right]$ is calculated by total energy consumption from multiple sources in a year divided by population (person) for Indonesia. $E_{avg}$ for the three years are shown by Table SI-A.1 and SI-A.2. Source of dataset is provided from Ministry of Energy and Mineral Resources Republic of Indonesia (2016, 2023).

**Table SI-A.1.** Energy source and $E_{avg}$ calculation for 2000 and 2009.

| Year | Energy source, final energy consumption by type (thousand BOE – Barrel of oil equivalent / year) | | | | | | | | Total energy (1,000 BOE/year) | Population (Thousand person) | $E_{avg} \left( W/person \right)$ |
| --- | --- | --- | --- | --- | --- | --- | --- | --- | --- | --- | --- |
|  | Biomass | Coal | Natural gas | Fuel | Other petroleum products | Briquette | LPG | Electricity |  |  |  |
| 2000 | 269,042 | 36,060 | 87,214 | 315,272 | 13,435 | 85 | 8,261 | 48,555 | 777,924 | 205,843 | 731 |
| 2009 | 279,169 | 82,587 | 118,587 | 335,271 | 55,663 | 220 | 24,384 | 82,499 | 978,380 | 234,757 | 809 |

**Table SI-A.2.** Energy source and $E_{avg}$ calculation for 2019.

| Year | Energy source, final energy consumption by type (thousand BOE – Barrel of oil equivalent / year) | | | | | | | | | | | | Total energy (1,000 BOE/year) | Population (Thousand person) | $E_{avg} \left( W/person \right)$ |
| --- | --- | --- | --- | --- | --- | --- | --- | --- | --- | --- | --- | --- | --- | --- | --- |
|  | Traditional biomass | Coal | Natural gas | Oil fuel | Industrial biomass | Briquette | LPG | Electricity | Biodiesel | Blending product | Bio gas | Gas oil |  |  |  |
| 2019 | 67,291 | 167,412 | 95,824 | 261,971 | 555 | 28 | 66,304 | 160,621 | 41,494 | 191,889 | 167 | 15,0395 | 1,203,951 | 268,075 | 871 |

Note: $1,000 BOE = 6.118\times{10}^{12} J$


**SI-B: Possible impacts of macro climate on LST**

In the conceptual framework of Lowry (1977), it is assumed that the three linear contributors make up the measured value of a variable ($V_{M}$) at a given point for atmospheric conditions or processes. The factors include (1) background condition ($V_{B}$) or macroclimate (global climate); (2) landscape or local climate ($V_{L}$), for example, from landscape or water bodies in the vicinity; and (3) human activities ($V_{H}$) such as anthropogenic heat release or urban effects, expressed as $V_{M} = V_{B} + V_{L} + V_{H}$. That framework is assumed to be applicable for surface conditions such as LST.


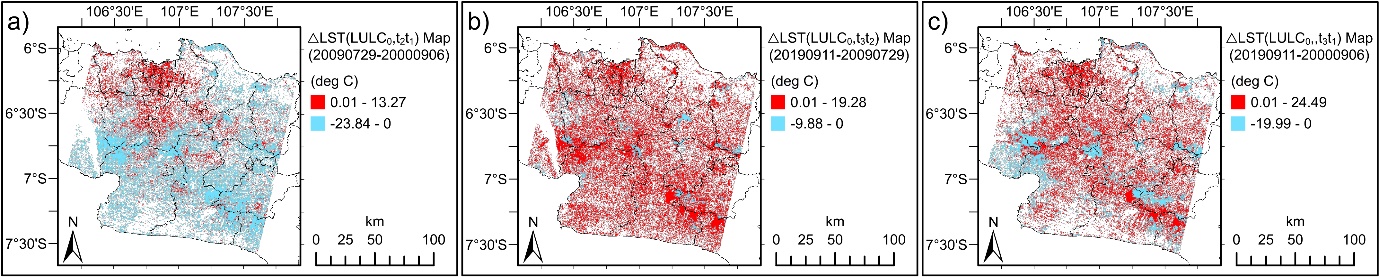


**Figure SI-B.1.** Spatial pattern of LST difference, $\Delta LST$, between two timestamps, 29 July 2009 and 6 September 2000 (left plot), 11 September 2019 and 29 July 2009 (middle plot), and 11 September 2019 and 6 September 2000 (right plot) of the unchanged LULC pixel, (LULC0), for the three timestamps.

Figure SI-B.1 shows the spatial patterns of the LST difference between two timestamps over pixels that the LULC did not change for the three time-steps, $LULC_{0}$, to minimize the effect of LULC conversion on LST. The intriguing spatial pattern is shown in Fig. SI-B.1.a, the LST difference between 29 July 2009 and 6 September 2000. Mostly, outside JMA, the negative values (light blue color) dominate, which means that the LST in 2009 is lower than in 2000. However, in JMA, positive values (red color) dominate. Based on the above-mentioned framework, if only $V_{B}$ applies (due to El Niño), the LST in 2009 should be lower than in 2000, but that did not happen in JMA. Then, if only $V_{H}$ applies (due to urbanization), similar features should happen in JMA, BC, and SKB. $V_{L}$ might have a contribution, but this discussion only focuses on macroclimate and urbanization.

Next, the mean surface sensible ($Q_{H}$) and latent ($Q_{E}$) heat fluxes are analyzed. $Q_{H}$ is the transfer of heat between the Earth’s surface and the atmosphere through the effects of turbulent air motion (but excluding any heat transfer resulting from condensation or evaporation). Meanwhile, $Q_{E}$ is the transfer of latent heat (resulting from water phase changes, such as evaporation or condensation) between the Earth’s surface and the atmosphere through the effects of turbulent air motion. The equation of surface energy balance (SEB) (Oke et al. 2017) shows that the change of surface temperature in time is partly contributed by $Q_{H}$ and $Q_{E}$. The other contributors are the change in net radiation ($Q^{*}$) and ground heat flux ($Q_{G}$).


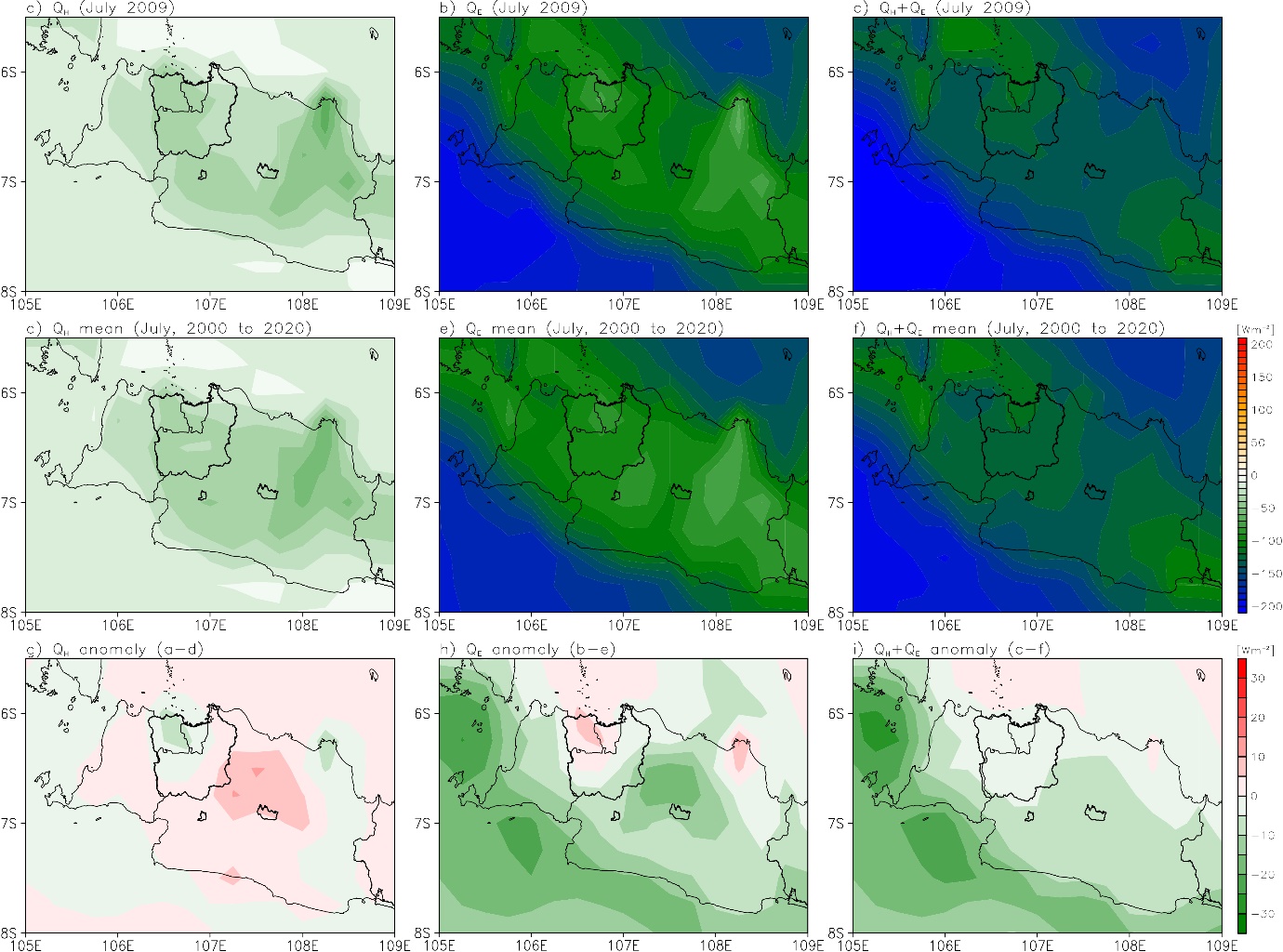


**Figure SI-B.2.** Monthly (first column) mean surface sensible heat flux (QH), (second column) mean surface latent heat flux (QE), and (third column) summation of both variables (QH + QE) for (first row) July 2009, (second row) long-term mean in July from 2000 to 2020, and (third row) the anomalies.

As shown in Fig. SI-B.2, the plots in the first row show (a) $Q_{H}$, (b) $Q_{E}$, and (c) summation of both variables in July 2009. The plots in the second row are similar to the first row except for the long-term mean (July, from 2000 to 2020) of those variables. While the third row shows the anomalies of each variable from the long-term mean. For the plots in the first and second rows, a negative (positive) value means the upward (downward) direction of the fluxes that will contribute to the decreasing (rising) of surface temperature.

From Fig. SI-B.2.g, in July 2009, the anomaly of $Q_{H}$ shows mostly positive anomalies over the mainland. It indicates less heat loss from the surface due to sensible heat flux. Meanwhile, from Fig. SI-B.2h, $Q_{E}$ in July 2009 has a majority of negative anomalies. It indicates more heat loss from the surface due to latent heat flux. It is noted that the anomalies of both variables around Jakarta and the northeast of west Java are different from the other regions in the mainland. In total, the anomaly of $Q_{H}+Q_{E}$ shows negative values but with a non-uniform spatial pattern, lower magnitude over the northern coast of the island. That indicates that more heat loses from the surfaces happened in the southern part of the study area (like BC and SKB). However, for JMA, the magnitude of the heat loss from surface is smaller. This discussion suggests that it is necessary to be careful in interpreting LST patterns of urban areas. The LST variation is not fully affected by urban growth, but also non-urbanization factors are embedded in the dataset.

**References**

Lowry, William P. 1977. “Empirical Estimation of Urban Effects on Climate: A Problem Analysis.” *Journal of Applied Meteorology and Climatology* 16(2):129–35. doi: https://doi.org/10.1175/1520-0450(1977)016<0129:EEOUEO>2.0.CO;2.

Ministry of Energy and Mineral Resources Republic of Indonesia. 2016. *Handbook of Energy & Economic Statistics of Indonesia, Final Edition*. Jakarta Pusat.

Ministry of Energy and Mineral Resources Republic of Indonesia. 2023. *Handbook of Energy & Economic Statistics of Indonesia*. Jakarta Pusat.

Oke, Timothy R., Gerald Mills, Andreas Christen, and James A. Voogt. 2017. *Urban Climates*.

1. * Corresponding authors. Address as above. E-mail addresses:

   d225973@hiroshima-u.ac.jp (Faiz Rohman Fajary)

   leehs@hiroshima-u.ac.jp (Han Soo Lee) [↑](#footnote-ref-1)
2. [↑](#footnote-ref-2)
